# Uræmic Poisoning

**Published:** 1872-05

**Authors:** A. Ford

**Affiliations:** Boone, Kent county, Michigan


ARTICLE VI. — *Uræmic Poisoning.* By A. FORD, M.D., Boone, Kent county, Michigan.

Feb. 11th, 1872. Mr. M. came into my office with his daughter 11 years old, for advice.

*Symptoms.* A slight puffiness of the face and some swelling of feet and ankles. Parents had noticed the swelling for a week. Child said she was not sick ; had no pain except occasionally a little in the region of the heart ; bowels costive, and urine scanty. Prescribed a cathartic, and a diuretic of acetate of potassa, which relieved the symptoms for the time.

On the morning of the 17th was summoned in great haste. C—— was having convulsions. Found the symptoms the same as at first, with entire suppression of urine, and patient vomiting every thing she swallowed. Spasm of both voluntary and involuntary muscles, lasting from three to five minutes, and recurring every half or three-fourths of an hour. Gave morphine, one-fourth grain, but it was immediately rejected ; used chloroform to control the spasms—it would stop them within about one minute. Stayed with the patient, and sent for Dr. M. for council. For three or four hours the patient was conscious between the fits, after that she was comatose.

Near night, Dr. M. came, and confirmed my diagnosis of poisoning from non-elimination, and accumulation of urea. Used morphine, one-fourth grain, by hypodermic injection, and repeated it in one hour and a half ; in three hours repeated it again. The vomiting having ceased, gave fluid ext. Aralia Hispida, dr. ss., once in two hours, and R. fluid ext. Verat. Viride, gtts. ij; fluid ext. Belladonna, gtts. j, in a teaspoonful of water once in two hours.

Interval between the convulsions increased to one hour and a half till ten o'clock, P. M., when they ceased. Continued same treatment through the night and next day. Toward evening found a fullness in the region of the bladder, and at seven o'clock, P. M., used the catheter and drew off twenty-four ounces of very dark-colored urine. During the night she passed urine three or four times, saturating a large folded sheet each time. Continued treatment, but only gave medicine half as often.

She remained unconscious for three days, and then the coma gradually left her. The dropsy subsided, and she convalesced finely.

